# Supplementary material for: The role of Patient and public involvement (PPI) in pre-clinical spinal cord research: An interview study
Source: PLoS One. 2024 Apr 29;19(4):e0301626. doi: 10.1371/journal.pone.0301626 (PMC11057720; doi:10.1371/journal.pone.0301626)
Supplement: S1 Appendix — (DOCX) [file pone.0301626.s001.docx]

**Appendix 1**

**Topic Guide**

Interviewer to welcome participant to the session, remind them that it is intended to be an informal discussion and their honest opinions (good or bad) are welcome.

Interviewer to invite questions, ensure the consent form has been completed, remind participant of confidentiality of data, and seek permission to start recording.

**Part A: Background/Personal Views**

1. Have you ever heard the term ‘patient and public involvement in research’ or ‘PPI’? What do you think it might mean?

Note 1: The interviewer will then tell the participant that ‘PPI’ will be used in the interview (this is suggested based on pilot participants’ feedback).

Note 2: If the participant does not know what it is or gives an incomplete/inaccurate answer, the INVOLVE definition will be provided:

*INVOLVE, a group that develop PPI policy, defines public involvement in research as research being carried out ‘with’ or ‘by’ members of the public rather than ‘to’, ‘about’ or ‘for’ them. This includes, for example:*

- *working with research funders to prioritise research;*
- *offering advice as members of a project steering group;*
- *commenting on and developing research materials;*
- *undertaking interviews with research participants.*

2. What is your opinion about PPI in research [based on this definition]?

3. Have you ever participated in a PPI activity? E.g. training, a workshop etc.?

- How did you come to be involved?
- What was it about?
- What did you think about it?

4. In your experience as a person affected by spinal cord injury, would you consider PPI in research important to you?

- Why/why not?
- How important is it for you to personally participate in patient and public involvement activities and why?

**Part B: The Spinal Cord Repair Project**

As explained in the participant information leaflet, the project aims to find new ways to repair spinal cord injury. This research is being conducted by research scientists in a laboratory setting, for example, on animal models or with human cells.

5. What should the goals of PPI for this research be from your perspective and why?

6. Some examples of times in research where patient and public involvement is used, include the following, what do you think is good/bad about each in the context of the spinal cord repair project and why?

- ‘Priority Setting’ – e.g. helping to identify research needs, ensure research is relevant to those affected by particular conditions, highlight potential future new directions
- ‘Management/oversight activities’ – e.g. sitting on project steering groups, or acting as a member of an advisory panel
- ‘Application and Design’ – e.g. review and write ‘lay material’ such as articles or blogs, design material for people affected by the condition
- ‘Dissemination/communicating research findings’ – e.g. identifying key findings to share with the public, helping to plan outreach/sharing activities for the public.

7. In what other ways might people affected by spinal cord injury be involved in your opinion?

8. In your opinion, what barriers might exist to involving people affected by spinal cord injury in PPI activities and how might they be overcome?

9. What would make it easier to involve people affected by spinal cord injury in PPI and why?

**C. Patient and Public Engagement**

‘Patient and Public Engagement’ or PPE is another way that people are included in research activities, often to allow sharing of information about completed research activities such as findings or results.

10. Is this important to you as a person affected by spinal cord injury? Why?

11. The following methods are commonly used to engage people affected by particular conditions with research.

What do you think is good or bad about the following options in terms of the spinal cord repair project and why?

- Social media (e.g. Twitter or blogging)
- Talks held in a college/university for the public
- Exhibitions at museums/discovery centres or festivals
- Science talks held in cafés or bars
- Media broadcasts (television or radio)
- Research-buddies (having a 1:1 relationship with a specific researcher)

12. Are there any other ways you think might be good ways to involve/engage people in spinal cord research?

**D. Closing**

13. Is there anything that we have not discussed that you would like to mention now?

14. What questions do you have for me?

Thank you for your time.
